# Cardiac Differentiation of Adipose Tissue-Derived Stem Cells Is Driven by BMP4 and bFGF but Counteracted by 5-Azacytidine and Valproic Acid 

**DOI:** 10.22074/cellj.2020.6582

**Published:** 2019-12-15

**Authors:** Sanaz Hasani, Arash Javeri, Asadollah Asadi, Masoumeh Fakhr Taha

**Affiliations:** 1 Department of Stem Cells and Regenerative Medicine, Institute for Medical Biotechnology, National Institute of Genetic Engineering tyand Biotechnology (NIGEB), Tehran, Iran; 2 Department of Biology, Faculty of Science, University of Mohaghegh Ardabili, Ardabil, Iran

**Keywords:** Adipose Tissue-Derived Stem Cells, Basic Fibroblast Growth Factor, BMP4, Cardiomyocyte, Small Molecules

## Abstract

**Objective:**

Bone morphogenetic protein 4 (BMP4) and basic fibroblast growth factor (bFGF) play important roles in embryonic heart development. Also, two epigenetic modifying molecules, 5ˊ-azacytidine (5ˊ-Aza) and valproic acid (VPA) induce cardiomyogenesis in the infarcted heart. In this study, we first evaluated the role of BMP4 and bFGF in cardiac trans-differentiation and then the effectiveness of 5´-Aza and VPA in reprogramming and cardiac differentiation of human adipose tissue-derived stem cells (ADSCs).

**Materials and Methods:**

In this experimental study, human ADSCs were isolated by collagenase I digestion. For cardiac differentiation, third to fifth-passaged ADSCs were treated with BMP4 alone or a combination of BMP4 and bFGF with or without 5ˊ-Aza and VPA pre-treatment. After 21 days, the expression of cardiac-specific markers was evaluated by reverse transcription polymerase chain reaction (RT-PCR), quantitative real-time PCR, immunocytochemistry, flow cytometry and western blot analyses.

**Results:**

BMP4 and more prominently a combination of BMP4 and bFGF induced cardiac differentiation of human ADSCs. Epigenetic modification of the ADSCs by 5ˊ-Aza and VPA significantly upregulated the expression of OCT4A, *SOX2, NANOG, Brachyury/T* and *GATA4* but downregulated *GSC* and *NES* mRNAs. Furthermore, pre-treatment with 5ˊ-Aza and VPA upregulated the expression of *TBX5, ANF, CX43* and *CXCR4* mRNAs in three-week differentiated ADSCs but downregulated the expression of some cardiac-specific genes and decreased the population of cardiac troponin I-expressing cells.

**Conclusion:**

Our findings demonstrated the inductive role of BMP4 and especially BMP4 and bFGF combination in cardiac trans-differentiation of human ADSCs. Treatment with 5ˊ-Aza and VPA reprogrammed ADSCs toward a more pluripotent state and increased tendency of the ADSCs for mesodermal differentiation. Although pre-treatment with 5ˊ-Aza and VPA counteracted the cardiogenic effects of BMP4 and bFGF, it may be in favor of migration, engraftment and survival of the ADSCs after transplantation.

## Introduction

Cardiovascular diseases are the most common causes of deaths worldwide (1). Despite the great advances in medical and surgical therapies, functional recovery of the infarcted heart remains elusive. A novel strategy for the treatment of advanced myocardial infarction is transplantation of stem cells or stem cell-derived cardiac progenitor cells into the damaged heart with the expectation that these cells can produce or stimulate generation of new cardiomyocytes and blood vessels in the injured tissue (2).

Adipose tissue has been considered as a valuable source of autologous mesenchymal stem cells for heart tissue engineering and cardiac repair. Beneficial role of adipose tissue-derived stem cells (ADSCs) in regeneration of ischemic heart disease is emanated from several properties, including differentiation to cardiomyocytes, endothelial cells and smooth muscle cells (3, 4), secretion of several angiogenic and anti-apoptotic factors (3, 5) and recruitment of endogenous stem cells into the damaged area (6). Although accumulating evidence has shown the capability of ADSCs for differentiation into cardiomyocytes and improvement of ventricular function in animal models of myocardial infarction (7-9), a highly efficient protocol for cardiac differentiation of human ADSCs is yet to be reported. Further studies are required to develop optimal media formulations which generate a large number of functional cardiomyocytes for embryology, toxicology, pharmacology and transplantation therapy purposes. In this regard, better understanding of the role of cardiogenic growth factors and small molecules which can reprogram somatic cells toward a more undifferentiated state is of utmost importance.

Bone morphogenetic protein 4 (BMP4) and basic fibroblast growth factor (bFGF) signaling play important roles in embryonic heart development (10, 11). A combination of bFGF and BMP2/4 has been shown necessary to induce Nkx2.5 expression and contractile phenotype in non-precardiac mesoderm of chicken embryos (12). In fact, BMPs and FGFs have complementary roles in cardiac development; BMP induces the specification of non-precardiac mesoderm cells to cardiac cell lineage, while FGF supports terminal differentiation of cardiomyocytes (13, 14).

5ʹ-azacytidine (5ʹ-Aza) and valproic acid (VPA) are two small molecules which regulate chromatin remodeling through inhibition of DNA methyltransferases and histone deacetylases, respectively (14). The positive role of 5ʹ-Aza and VPA in cardiac differentiation has been demonstrated by different groups (14-16), although contradictory results have also been reported (17, 18). In an attempt by Thal and colleagues (14), treatment of the endothelial progenitor cells with 5ʹ-Aza and VPA significantly upregulated the expression of pluripotency and cardiacspecific genes and increased the cardiogenic potential of the reprogrammed cells. However, this should be kept in mind that reactivation of previously silent genes by epigenetic modifiers like 5ʹ-Aza and VPA is not limited to pluripotency-associated or cardiacspecific genes but is rather indicative of a global gene transcription. So, an appropriate culture condition is necessary to direct the fate of reprogrammed cells towards a cardiogenic lineage (14).

Despite the available evidence demonstrating the inductive role of BMP4 and bFGF growth factors (10- 12) as well as small molecules like 5ʹ-Aza and VPA (14- 16) in cardiac differentiation, there is no report regarding the impact of these factors on cardiac differentiation of hADSCs. We previously showed that BMP4 treatment induces the expression of cardiac-specific markers in mouse ADSCs (19). In the current study, we first evaluated the role of BMP4 individually, and then in combination with bFGF in cardiac trans-differentiation of human ADSCs and finally examined the impact of 5ʹ-Aza and VPA on reprogramming and cardiac differentiation of the ADSCs.

## Materials and Methods

### Isolation and culture of human adipose tissue-derived stem cells

In this experimental study, adipose tissue samples were harvested from five 40-45 years old women undergoing elective abdominoplasty after obtaining informed consent. The study was approved by the Ethics Committee of National Institute of Genetic Engineering and Biotechnology (7-8-93/NIGEB).

Isolation and characterization of the ADSCs was performed as described previously (20). Briefly, adipose tissue was minced and digested by 2 mg/ml collagenase I (Thermo Fisher Scientific, USA) in PBS containing 2% bovine serum albumin (BSA, Sigma Aldrich, USA). The stromal vascular fraction (SVF) was plated at 5×10^4^ cells/ ml in tissue culture flasks. Growth medium contained Dulbecco’s Modified Eagle’s Medium (DMEM), 20% fetal bovine serum (FBS), 100 U/ml penicillin and 100 µg/ml streptomycin (all from Gibco, Thermo Fisher Scientific, USA). Medium was changed every other day, and the cells were subcultured after reaching 80-90% confluency.

### Cardiac differentiation of human adipose tissuederived stem cells

For cardiac differentiation, third to fifth-passaged ADSC were seeded into 0.1% gelatin-coated 6-well tissue culture plates with a density of 10^5^ cell/ml (2 ml per each well). After 24 hours, the cells were induced for cardiac differentiation with 10 ng/ml bFGF (Sigma-Aldrich, USA) and 20 ng/ml BMP4 (Thermo Fisher Scientific, USA) for four days. After the induction stage, growth factors were omitted completely and differentiation of the cells was continued in 10% FBS-containing medium up to three weeks. The ADSCs that were cultured in the same medium without bFGF and BMP4 treatment were used as the control group.

To investigate the impact of DNA methyltransferase and histone deacetylase inhibitors on cardiac differentiation of ADSCs, the cells were pre-treated with 10 µM 5ʹ-Azacitidine (Sigma-Aldrich, USA) and 500 nM VPA (Sigma-Aldrich, USA) for 24 hours and then were treated with 10 ng/ml bFGF and 20 ng/ml BMP4 as described above.

### Gene expression analysis

Total RNAs were extracted from three-week differentiated ADSCs using High Pure RNA Isolation Kit (Roche Applied Science, Germany). Briefly, cDNA was synthesized from 1 µg of total RNA using cDNA Synthesis Kit (Thermo Fisher Scientific, USA). PCR amplification on the cDNA samples was performed using PCR master mix (Ampliqon, Denmark) and specific primers, as described in Table S1 (See Supplementary Online Information at www. celljournal.org).

RealQ PCR Master (Ampliqon, Denmark) were used for quantitative assessment of gene expression by realtime polymerase chain reaction (qPCR) on a RotorGene^TM^ 6000 (Corbett Research, Australia) real-time analyzer. β2 microglobulin (*B2M*) and β-actin (*ACTB*) were used as the internal reference genes. The size of the qPCR products were assessed both by melting curve analysis and also by agarose gel electrophoresis.

Comparative quantification was performed using REST 2009 (Relative Expression Software Tool, Qiagen) based on Pair Wise Fixed Reallocation Randomization Test^®^ (21). At least, three biological replicates of each group were included in the qPCR experiments, and *B2M* and *ACTB* were used to normalize the quantitative data.

### Immunocytochemistry

Since cell density of three-week differentiated ADSCs was too high, the cells were dissociated using trypsin-EDTA (Gibco, USA) and cultured at half the density in gelatin-coated 4-well tissue culture plates. After 24 hours, the cells were fixed using 4% paraformaldehyde and permeabilized with 0.2% Triton X-100. 10% goat serum was used to block non-specific binding sites. Next, the cells were incubated with the primary monoclonal antibodies against α-actinin (Sigma-Aldrich, USA) and cardiac troponin I (Santa Cruz Biotechnology, USA) and then with goat antimouse FITC-conjugated IgG (Sigma-Aldrich, USA). The stained cells were observed by a fluorescence microscope (Nikon, Japan).

### Flow cytometry analysis

Three-week differentiated cells were dissociated using trypsin-EDTA and fixed in cold 70% ethanol. The cells were permeabilized with 0.2% Triton X-100. After washing, the cells were incubated with the primary antibody against cardiac troponin I (Santa Cruz Biotechnology, USA) and then with fluorescein isothiocyanate (FITC)-conjugated goat anti-mouse IgG (Sigma-Aldrich, USA). Some cells were only stained with the secondary antibody and were used as the negative control. Flow cytometry was performed using an Attune® Acoustic Focusing Cytometer (Applied Biosystems, Thermo Fisher Scientific, USA). FlowJo vX.0.6 software (Tree Star Inc., Ashland, USA) was used for analysis of the results.

### Western blot analysis

For protein analysis by western blot, three-week differentiated ADSCs were homogenized in ice-cold Radioimmunopercipitation assay (RIPA) lysis buffer and were centrifuged at 13000 g for 15 minutes at 4˚C. After collecting the supernatant, protein concentration was determined by Bradford assay. For each sample, 50 µg of protein was separated using sodium dodecyl sulphate-polyacrylamide gel electrophoresis (SDSPAGE) and transferred to polyvinylidene difluoride (PVDF, Roche) membranes. Blocking of non-specific binding sites was achieved by 5% non-fat dried milk in Tris-buffered saline containing 0.1% Tween-20 (TBST). After blocking, the membranes were incubated with the diluted primary antibodies against glyceraldehyde 3-phosphate dehydrogenase (GAPDH, Sigma-Aldrich, G8795), α-actinin (Sigma-Aldrich, USA), desmin (Sigma-Aldrich, USA) and connexion 43 (Sigma-Aldrich, USA) overnight at 4˚C. Then, the membranes were incubated with goat anti-mouse horseradish peroxidase (HRP)-conjugated secondary IgG for 1 hour at room temperature. Enhanced chemiluminescence (ECL) kit (Najm Biotech Co., Iran) was used to detect the immunoreactive bands.

## Results

### Isolation, culture and differentiation of human adipose tissue-derived stem cells

**Fig 1:**
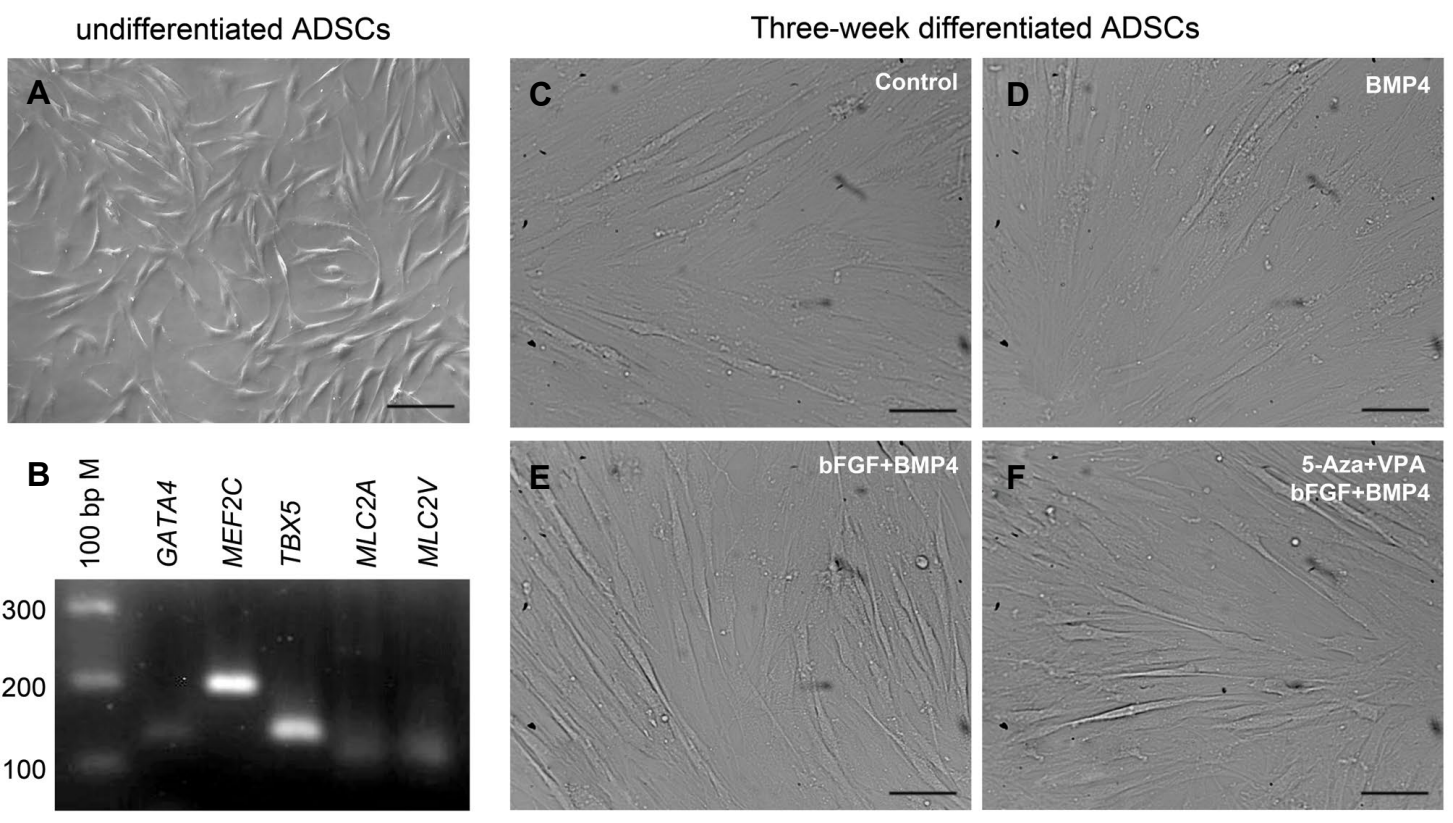
The undifferentiated and three-week differentiated ADSCs. **A.** Third-passaged ADSCs showed a fibroblast-like morphology, **B.** Expressed some cardiac-specific genes, **C.** After cardiac differentiation in the control (no treatment),** D.** BMP4 alone, **E,** and **F.** A combination of bFGF and BMP4 with or without pre-treatment with 5-Aza and VPA, differentiating ADSCs showed an elongated morphology (scale bar: 50 µm). ADSCs; Adipose tissue-derived stem cells, BMP4; Bone morphogenetic protein 4, bFGF; basic fibroblast growth factor, 5ˊ-Aza; 5ˊ-azacytidine, and VPA; Valproic acid (scale bar: 50 µm).

Within 3-4 hours, the ADSCs attached to the growth surfaces of tissue culture plates. The ADSCs proliferated rapidly and were passaged 2-3 times a week. The undifferentiated cells showed a fibroblastlike morphology (Fig .1A). The mesenchymal stem cell feature and multipotential differentiation capability of the ADSCs was determined as described previously by our team (20, 22, 23). Third-passaged ADSCs expressed cardiac transcription factors, *GATA4, MEF2C* and TBX5, and cardiac-specific genes, *MLC2A/MYL7* and *MLC2V/MYL2* (Fig .1B) which possibly indicate the capability of these cells for cardiac differentiation.

After three weeks cardiac differentiation in different experimental groups, including control (no treatment, Fig .1C), BMP4 alone (Fig .1D) and a combination of bFGF and BMP4 with or without pre-treatment with 5-Aza and VPA (Fig .1E, F, respectively), differentiating ADSCs showed an elongated morphology.

### BMP4 induces cardiac trans-differentiation of human adipose tissue-derived stem cells

**Fig 2:**
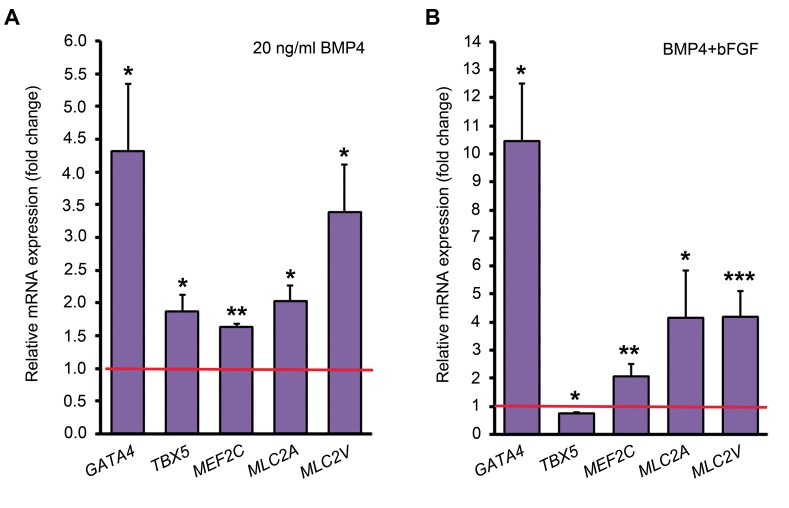
Quantitative analysis of some cardiac-specific genes by comparative method. **A.** The expression level of each gene in the control group (untreated ADSCs) has been assumed 1 (indicated by the red line) and its expression in BMP4 treatment group was compared to that and **B.** The expression level of each gene in the BMP4 treatment group has been assumed 1 (indicated by the red line) and its expression in BMP4 plus bFGF treatment group was compared to that. *; P<0.05, **; P<0.01, ***; P<0.001 (Pair Wise Fixed Reallocation Randomization Test^®^ performed by REST 2009 software), ADSCs; Adipose tissue-derived stem cells, BMP4; Bone morphogenetic protein 4, and bFGF; Basic fibroblast growth factor.

We previously showed that BMP4 induces the expression of cardiac-specific genes in mouse ADSCs (19). In the current study, treatment of human ADSCs with 20 ng/ ml BMP4 upregulated the expression of *GATA4, TBX5, MEF2C, MLC2A* and *MLC2V* mRNAs by 4.31, 1.88, 1.63, 2.03 and 3.4 folds compared to the control group, respectively (Fig .2A).

### A combination of BMP4 and bFGF augments cardiac trans-differentiation of human ADSCs

To investigate the synergistic effect of BMP4 and bFGF in cardiac differentiation of human ADSCs, third to fifth-passaged ADSCs were simultaneously treated with 10 ng/ml bFGF and 20 ng/ml BMP4 for the first four days of differentiation. The expression levels of *GATA4, MEF2C, MLC2A* and *MLC2V* mRNAs were upregulated respectively by 10.46, 2.09, 4.16 and 4.21 folds in the BMP4 and bFGF combination treatment group compared to the BMP4 treatment alone (Fig .2B).

### Treatment of the ADSCs with 5ʹ-Aza and VPA upregulated the expression of some pluripotency and mesodermal genes

**Fig 3:**
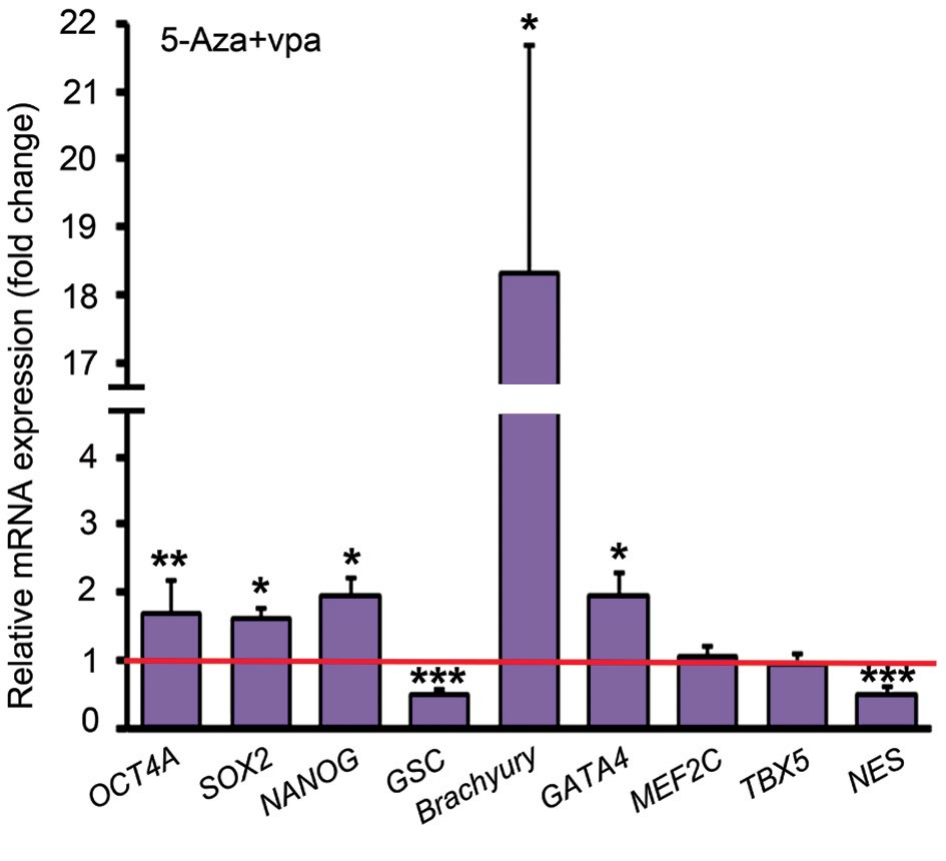
Quantitative analysis of some genes involved in the maintenance of pluripotency or early development by comparative method; the expression level of each gene in the control ADSCs has been assumed 1 (indicated by the red line) and its expression in the ADSCs treated with 5ʹ-Aza and VPA was compared to that. *; P<0.05, **; P<0.01, ***; P<0.001 (Pair Wise Fixed Reallocation Randomization Test® performed by REST 2009 software), ADSCs; Adipose tissue-derived stem cells, 5ˊ-Aza; 5ˊ-azacytidine, and VPA; Valproic acid.

24 hours treatment of the undifferentiated ADSCs with 5ʹ-Aza and VPA upregulated the expression of *OCT4A*, *SOX2* and *NANOG* by 1.69 (P=0.009), 1.6 (P=0.015) and 1.93 folds (P=0.029), respectively. Goosecoid (*GSC*) and Nestin (*NES*) were downregulated by half after 5ʹ-Aza and VPA treatment. *Brachyury/T* and *GATA4* expression in the ADSCs treated with 5ʹ-Aza and VPA were respectively 18.33 folds and 1.95 folds higher than the untreated ADSCs, while the expression of *MEF2C* and *TBX5* was not changed significantly (Fig .3).

### Pre-treatment with 5ʹ-Aza and VPA affected cardiac differentiation of human ADSCs

**Fig 4:**
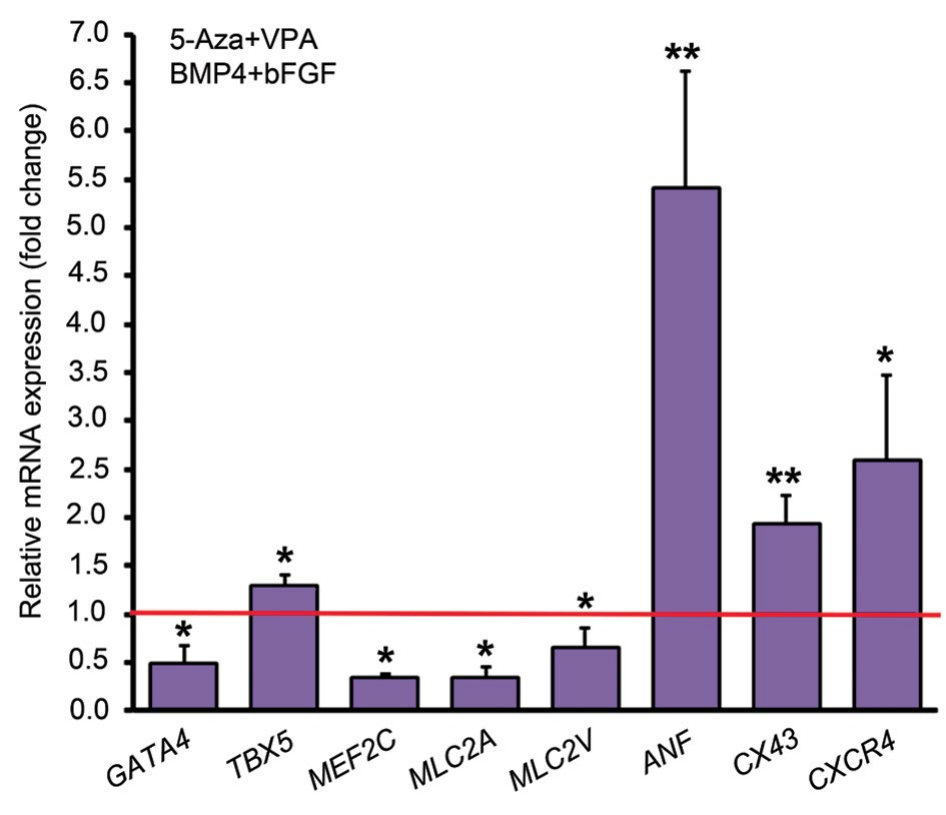
Quantitative analysis of some cardiac-specific genes by comparative method. The expression of each gene in the group with BMP4 and bFGF treatment but without 5-Aza and VPA pretreatment has been assumed 1 (indicated by the red line) and gene expression levels in the group with 5-Aza and VPA pre-treatment was compared to that. *; P<0.05, **; P<0.01 (Pair Wise Fixed Reallocation Randomization Test® performed by REST 2009 software). BMP4; Bone morphogenetic protein 4, bFGF; basic fibroblast growth factor, 5ˊ-Aza; 5ˊ-azacytidine, and VPA; Valproic acid.

To elucidate the influence of DNA methyltransferase and histone deacetylase inhibitors on cardiac differentiation of human ADSCs, the cells were pretreated with 10 µM 5ʹ-Aza and 0.5 µM VPA for 24 hours and then were induced with 10 ng/ml bFGF and 20 ng/ml BMP4 in 10% FBS-containing medium. As revealed by qPCR analysis, pre-treatment with 5ʹ-Aza and VPA downregulated the expression of *GATA4, MEF2C, MLC2A* and *MLC2V* by mean factors of 0.5, 0.35, 0.34 and 0.66, respectively. However, *TBX5, ANF, CX43* and *CXCR4* were upregulated after pretreatment with 5-Aza and VPA by 1.3, 5.4, 1.94 and 2.6 folds, respectively (Fig .4).

### Protein expression analysis

**Fig 5:**
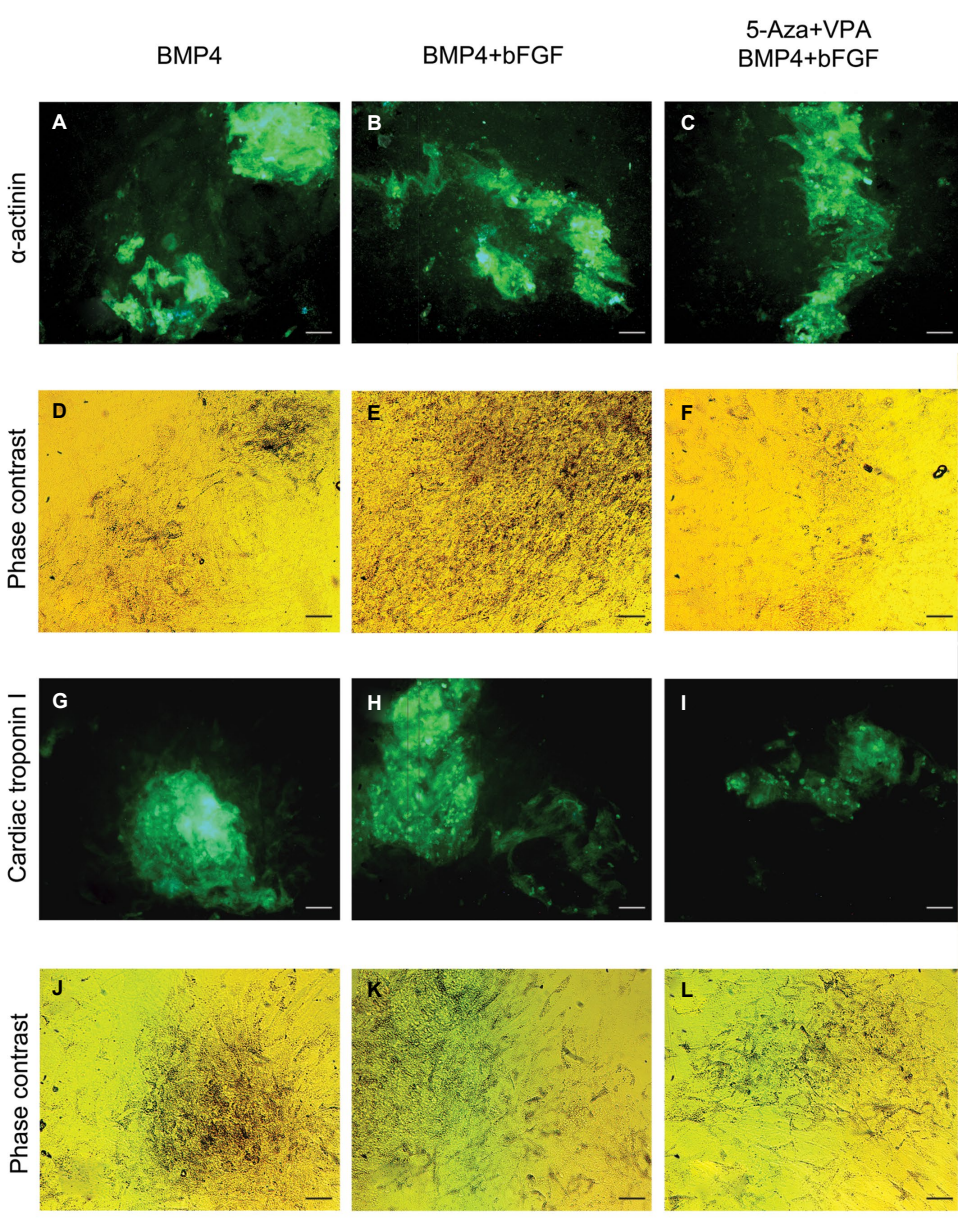
Immunocytochemical staining of three-week differentiated ADSCs. **A-C.** Immunostaining for α-actinin in the BMP4 treatment group and the groups which received a combination of BMP4 and bFGF with or without 5ʹ-Aza and VPA pre-treatment, **D-F.** Phase contrast images of A to C, respectively, **G-I.** Immunostaining for cardiac troponin I in the BMP4 treatment group and the groups which received a combination of BMP4 and bFGF with or without 5ʹ-Aza and VPA pre-treatment, and **J-L.** Phase contrast images of G to I, respectively (scale bar: 50 µm).

BMP4 treatment group and the groups which received a combination of BMP4 and bFGF with or without 5ʹ-Aza and VPA pre-treatment were assessed for the expression of α-actinin and cardiac troponin I as two cardiac-specific proteins. As revealed by immunocytochemistry, after trypsinization and re-plating the differentiated cells, ADSC-derived cardiomyocyte-like cells tend to form aggregations which showed positive immunostaining for α-actinin (Fig .5A-F) and cardiac troponin I (Fig .5G-L) proteins.

**Fig 6:**
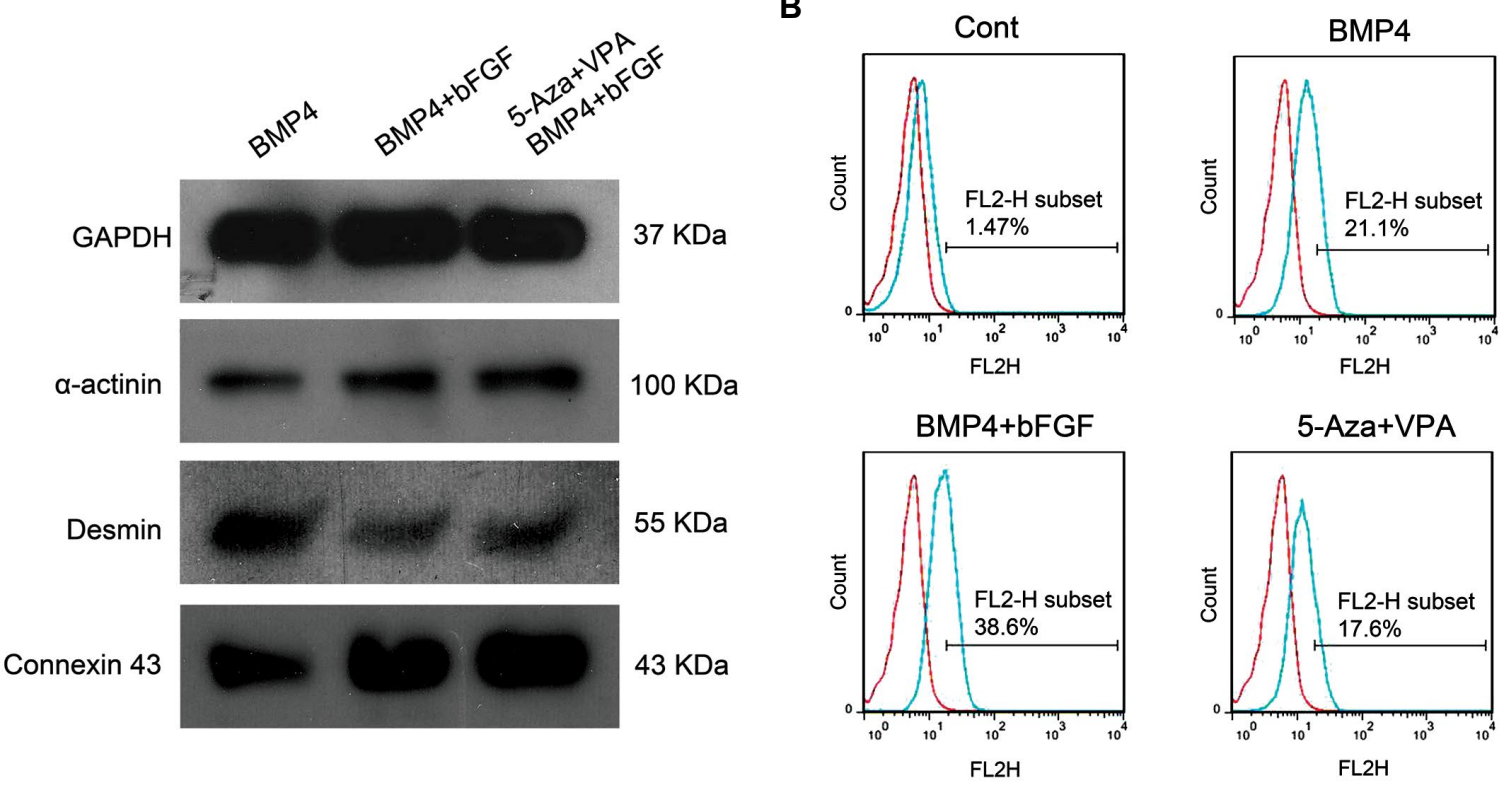
Western blot and flow cytometry analyses. **A.** Western blot analysis for the expression of α-actinin, desmin and connexin 43 proteins and **B.** Flow cytometry analysis for the expression of cardiac troponin I protein in three-week differentiated ADSCs of the control group, BMP4 treatment group and the groups which received a combination of BMP4 and bFGF with or without 5ʹ-Aza and VPA pre-treatment. ADSCs; Adipose tissue-derived stem cells, BMP4; Bone morphogenetic protein 4, bFGF; basic fibroblast growth factor, 5ˊ-Aza; 5ˊ-azacytidine, and VPA; Valproic acid.

Western blot analysis demonstrated the expression of α-actinin, desmin and connexin 43 proteins in the differentiated cells. α-actinin and connexin 43 showed their maximum expression in the cells pre-treated with 5-Aza and VPA and followed by BMP4 and bFGF treatment (Fig .6A). Based on flow cytometry analysis, about 21% of the cells in the BMP4 treatment group, 39% of the cells which treated with BMP4 and bFGF combination without 5-Aza and VPA pre-treatment and 18% of the cells pre-treated with 5-Aza and VPA and induced with BMP4 and bFGF combination showed positive staining for cardiac troponin I protein. In the control group, about 1.5% of the cells expressed cardiac troponin I protein (Fig .6B).

## Discussion

In the current study, we first examined the influence of BMP4 on cardiomyocyte trans-differentiation of human ADCSs. BMPs are members of TGFβ superfamily with essential roles in both mesoderm induction and embryonic heart development (11). While increasing evidence support the inductive role of BMPs in cardiac differentiation, some studies point to the temporally and spatially regulated expression of BMPs and BMP antagonists during heart development (24). BMP2 and BMP4 inhibit cardiomyogenesis during gastrula stage of chicken embryos (25). In mouse, noggin show a transient but strong expression in the anterolateral plate mesoderm and has a critical role in cardiac differentiation (26). Similar contradictory results have been obtained during cardiac differentiation of embryonic and adult stem cells. As reported by Yuasa et al. (26), inhibition of BMP signalling in a period between the undifferentiated state and early phase of embryoid body formation increases the incidence of beating EBs and the expression of cardiac transcription factors. We showed previously that BMP4 treatment inhibits cardiac differentiation of mouse embryonic stem cells (ESCs) in serum-containing media (27), although the complete removal of serum is not in favour of cardiomyocyte development (28). Some other investigators have demonstrated the inductive role of BMP4 in cardiac differentiation of human ESCs in a serum-based condition (29). Treatment of human bone marrow-derived mesenchymal stem cells (BM-MSCs) with BMP4 shifts the fate of cells toward a cardiac phenotype rather than the skeletal-like myocytes (30). We previously showed that BMP4 treatment of mouse ADSCs, especially in a knockout serum replacement (KoSR)-containing medium, induces the expression of cardiac-specific markers (19). In the current study, we examined the effect of BMP4 on cardiac differentiation of human ADSCs and showed that treatment of the ADSCs with 20 ng/ml BMP4 increases the expression of *GATA4, MEF2C, TBX5, MLC2A* and *MLC2V* mRNAs.

bFGF is a paracrine FGF with significant roles in development and pathophysiology of the heart (10). Barron et al. (12) showed that treatment of non-precardiac mesoderm of stage 6 chicken embryos with a combination of bFGF and BMP2/4 is necessary to induce Nkx2.5 expression and to promote contractile phenotype. In fact, both BMPs and FGFs act as cardiac specification factors; BMP specifies non-precardiac mesoderm cells to cardiac lineage (12), while FGF functions as a survival factor and supports their terminal differentiation (13). Here we examined the role of bFGF-BMP4 combination in cardiac differentiation of human ADSCs and showed that except for *TBX5* all tested cardiac markers, including *GATA4, MEF2C, MLC2A* and *MLC2V* mRNAs and CX43 and α-actinin proteins, were upregulated. Also, combined application of bFGF-BMP4 increased the population of cardiac troponin I-expressing cells to about 37% compared to 21% in BMP4 treatment alone.

In this study, *TBX5* was downregulated in the ADSCs treated with bFGF-BMP4 combination compared to BMP4 alone. In human, *TBX5* transcription factor is expressed in all developing heart chambers, but its expression in the atria is significantly higher than the ventricles (31). Also, ventricular expression of *TBX5* decreases at late embryonic stage and after birth (32). The known target genes for *TBX5* are atrial natriuretic factor (ANF) and connexin 40 (CX40) which are normally expressed in the atria and trabeculae (33). Therefore, lower expression of *TBX5* in the cells treated with bFGF-BMP4 combination than the BMP4-treated cells may be due to a reduction in atrial specification of myocytes.

Previous human clinical trials demonstrate the safety and efficacy of ADSCs for regeneration of myocardial infarction (34). However, a significant portion of this reparative function is emanated from secretion of several angiogenic and anti-apoptotic factors (3, 5) and recruitment of endogenous stem cells into the injury site (6). ADSCs rarely differentiate into cardiomyocytes in vivo (35), and even when collected from aged patients, they have a diminished capability for proliferation and differentiation (36). Epigenetic modification of ADSCs by small molecules may reprogram ADSCs towards a more pluripotent state, enhance their functional properties and improve their functionality after transplantation. In this study, we examined effectiveness of two epigenetic modifying molecules, 5ʹ-Aza and VPA, for reprogramming of human ADSCs towards a more undifferentiated state. 5ʹ-Aza and VPA, which are inhibitors of DNA methyltransferases and histone deacetylases respectively, have been used in generation of induced pluripotent stem cells (iPSCs) to improve reprogramming efficiency (37).

24 hours treatment of the undifferentiated ADSCs with a combination of 5ʹ-Aza and VPA upregulated the expression of some pluripotency transcription factors, including *OCT4A, SOX2* and *NANOG*. Also, treatment of the ADSCs with 5ʹ-Aza and VPA resulted in downregulation of definitive endoderm marker *GSC* and early neuroectoderm marker NES, and upregulation of mesendodermal marker *Brachyury/T* and cardiac transcription factor *GATA4*. Altogether, these findings suggest a delicate alteration in gene expression profile of the ADSCs and tendency of the reprogrammed cells for differentiation towards mesodermal lineages.

We assessed the influence of 5ʹ-Aza and VPA on cardiac differentiation of human ADSCs. It has been shown that both chemical factors remodel chromatin to allow expression of transcriptionally inactivated genes and to induce differentiation toward cardiomyocytes (15, 16). In 2012, Thal et al. (14) showed that epigenetic reprogramming of endothelial progenitor cells with 5ʹ-Aza and VPA improves repair of infarcted hearts by both cardiomyogenesis and vascularization. In contrast, we showed here that pre-treatment with a combination of 5ʹ-Aza and VPA downregulated the expression of *GATA4, MEF2C, MLC2A* and *MLC2V* which indicates the suppressive impact of these combination on cardiac differentiation of human ADSCs. Flow cytometry analysis for cardiac troponin I protein supports this conclusion since the population of immunostained cells decreased from 39% in the group which only received BMP4 and bFGF combination to about 18% in the BMP4 and bFGF combination group with 5ʹ-Aza and VPA pre-treatment. Of course, these findings do not contradict the stimulatory role of 5ʹ-Aza and VPA on cardiac differentiation and may just reflect the consequence of using these two agents at the same time. Perhaps, if the cells were initially treated with VPA for 24 hours and then with 5ʹ-Aza for 24 hours, as shown by Thal et al. (14), this might have a positive effect on cardiac differentiation. The other concentrations of these two small molecules can also be tested. However, pre-treatment with 5ʹ-Aza and VPA upregulated the expression of *TBX5, ANF* and *CX43* mRNAs and CX43 and α-actinin proteins. The reason for this discrepancy in the expression of cardiac-specific genes is not clear, but it is interesting to note that not only the expression of *ANF* is regulated by *TBX5* (33) but also *CX43* has been identified as a target for *TBX* factors (38). So, the simultaneous increase in the expression of these three genes is not far from the mind. Altogether, the upregulated expression of TBX5 and ANF genes may be due to an increased differentiation of atrial myocytes after 5ʹ-Aza and VPA pre-treatment. On the other hand, previous studies have demonstrated that *CX43* increases the survival of MSCs after transplantation into the ischemic heart and so may improve therapeutic efficacy of transplanted cells (9).

Stromal cell-derived factor (SDF)-1 and its membrane receptor, CXCR4, play pivotal roles in the migration, homing and engraftment of multiple stem cell types. At the injury site, SDF-1 expression increases and recruits circulating CXCR4-expressing MSC. Strategies to induce CXCR4 upregulation increases the migration and engraftment of MSCs *in vivo* (39). In the present study, pre-treatment with a combination of 5ʹ-Aza and VPA significantly upregulated the expression of CXCR4 in the ADSCs. This finding is in agreement with previous studies showing that 5-Aza and VPA significantly increase *CXCR4* expression in other types of stem cells (40).

## Conclusion

Our findings demonstrated that cardiac differentiation of human ADSCs can be induced by BMP4 but more significantly by a combination of BMP4 and bFGF. Treatment of the ADSCs with a combination of 5ʹ-Aza and VPA, which are respectively DNA methyltransferase and histone deacetylase inhibitors, significantly upregulated the expression of pluripotency transcription factors which indicates reprogramming of the ADSCs towards a more undifferentiated state. Downregulation of GSC and NES and upregulation of Brachyury/T and *GATA4* mRNAs in the ADSCs treated with 5ʹ-Aza and VPA suggests improved potential of the reprogrammed cells for mesodermal differentiation. However, pre-treatment with 5ʹ-Aza and VPA compromised the cardiogenic effects of BMP4 and bFGF which was determined by downregulation of some cardiac-specific genes and a decrease in the population of cardiac troponin I-expressing cells. Nevertheless, 5ʹ-Aza and VPA upregulated the expression of *TBX5, ANF, CX43* and *CXCR4* mRNAs which may improve migration, engraftment and survival of the ADSCs after transplantation into the injury site.

## Supplementary PDF


