# Supplementary material for: Cardiac Differentiation of Adipose Tissue-Derived Stem Cells Is Driven by BMP4 and bFGF but Counteracted by 5-Azacytidine and Valproic Acid
Source: Cell J. 2019 Dec 15;22(3):273–82. doi: 10.22074/cellj.2020.6582 (PMC6947007; doi:10.22074/cellj.2020.6582)
Supplement: Supplementary file 1 [file Cell-J-22-273-s01.pdf]

CELL JOURNAL (Yakhteh)

Supplementary Information for

Cardiac Differentiation of Adipose Tissue-Derived Stem Cells Is Driven by BMP4 and bFGF but Counteracted by 5-Azacytidine and Valproic Acid

Sanaz Hasani, M.Sc.[1, 2], Arash Javeri, M.D., Ph.D.[1], Asadollah Asadi, Ph.D.[2], Masoumeh Fakhr Taha, Ph.D.[1*]

1. Department of Stem Cells and Regenerative Medicine, Institute for Medical Biotechnology, National Institute of Genetic Engineering and Biotechnology (NIGEB), Tehran, Iran
2. Department of Biology, Faculty of Science, University of Mohaghegh Ardabili, Ardabil, Iran

*Corresponding Address: P.O.Box: 14965-161, Department of Stem Cells and Regenerative Medicine, Institute for Medical Biotechnology, National Institute of Genetic Engineering and Biotechnology (NIGEB), Tehran, Iran
Email: mftaha@nigeb.ac.ir

Table S1: Primers used for reverse transcription-polymerase chain reaction (RT-PCR) and quantitative real-time PCR

Gene	Forward	Reverse	Size (bp)	Accession
ACTB	CCTGGGCATGGAGTCCTGT	ATCTCCTTCTGCATCCTGTCG	153	NM_001101
B2M	TCCAGCGTACTCCAAAGATTCA	GTCAACTTCAATGTCGGATGGAT	113	NM_004048
OCT4A	GATGTGGTCCGAGTGTGGTT	AAGGGACCGAGGAGTACAGT	202	NM_002701
NANOG	GAAGCATCCGACTGTAAAGAATC	TGGTGGAAGAATCAGGGCTGT	169	NM_024865
SOX2	AGAACCCCAAGATGCACAACT	TCCTTCTTCATGAGCGTCTTG	184	NM_003106
GSC	GAAAGTGGAGGTCTGGTTTAA	TACCTTCCTCTTCCCTCTTCT	143	NM_173849
Brachyury/*T*	CTCCAACCTATTCTGACAACTC	ACTGACTGGAGCTGGTAGGT	141	NM_003181
NES	ACCTCAAGATGTCCCTCAGC	TCAGGACTGGGAGCAAAGATC	186	NM_006617
GATA4	CTGTGCCCGTAGTGAGATGA	ACCAGCAGCTCCTTCAGGCA	141	NM_002052
MEF2C	AGACCACCTGTGTTACCTGCA	AGATTGTGTGCTGTTCCACCT	203	NM_002397
TBX5	CCCGTCACAGACCATTTATTATC	AAGGCTGGAAGGCGGATGTTT	143	NM_000192
MLC2A	GGTCTGTCCCATTGAGCTTCT	AGCTGGGGAAGGTGAGTGT	120	NM_021223
MLC2V	GGTCCGCTCCCTTAAGTTTCT	CTTGGGCGAGTGAACGTGAA	118	NM_000432
ANF	CGCTTCTTCATTCGGCTCAC	GGATTTCAAGAATTTGCTGGACC	97	NM_006172
